# Effectiveness of an online module: climate-change and sustainability in clinical practice

**DOI:** 10.1186/s12909-022-03734-8

**Published:** 2022-09-17

**Authors:** H. Dunne, C. Rizan, A. Jones, M. F. Bhutta, T. Taylor, S. Barna, C. J. Taylor, M. Okorie

**Affiliations:** 1 Cambridge University Hospital, Cambridge, UK; 2 University Hospitals Sussex NHS Foundation Trust & Brighton and Sussex Medical School, Brighton, UK; 3 Brighton and Sussex Medical School, Brighton, BN1 9PX UK; 4 Brighton and Sussex Medical School & University Hospitals Sussex NHS Foundation Trust GB, Brighton, UK; 5 University Hospitals Southampton, Southampton, SO16 6YD UK; 6 Centre for Sustainable Healthcare, 291, Cranbrook house, 287 Bambury Rd, Summertown, Oxford, OX2 7JQ England

**Keywords:** Sustainability, Climate-change, Online, Multimedia

## Abstract

**Background:**

Climate change has significant implications for health, yet healthcare provision itself contributes significant greenhouse gas emission. Medical students need to be prepared to address impacts of the changing environment and fulfil a key role in climate mitigation. Here we evaluate the effectiveness of an online module on climate-change and sustainability in clinical practice designed to achieve learning objectives adapted from previously established sustainable healthcare priority learning outcomes.

**Methods:**

A multi-media, online module was developed, and 3^rd^ and 4^th^ year medical students at Brighton and Sussex Medical School were invited to enrol. Students completed pre- and post-module questionnaires consisting of Likert scale and white space answer questions. Quantitative and qualitative analysis of responses was performed.

**Results:**

Forty students enrolled and 33 students completed the module (83% completion rate). There was a significant increase in reported understanding of key concepts related to climate change and sustainability in clinical practice (*p* < 0.001), with proportion of students indicating good or excellent understanding increasing from between 2 – 21% students to between 91 – 97% students. The majority (97%) of students completed the module within 90 min. All students reported the module was relevant to their training. Thematic analysis of white space responses found students commonly reported they wanted access to more resources related to health and healthcare sustainability, as well as further guidance on how to make practical steps towards reducing the environmental impact within a clinical setting.

**Conclusion:**

This is the first study to evaluate learner outcomes of an online module in the field of sustainable health and healthcare. Our results suggest that completion of the module was associated with significant improvement in self-assessed knowledge of key concepts in climate health and sustainability. We hope this approach is followed elsewhere to prepare healthcare staff for impacts of climate change and to support improving the environmental sustainability of healthcare delivery.

**Trial registration:**

Study registered with Brighton and Sussex Medical School Research Governance and Ethics Committee (BSMS RGEC). Reference: ER/BSMS3576/8, Date: 4/3/2020.

**Supplementary Information:**

The online version contains supplementary material available at 10.1186/s12909-022-03734-8.

## Background

Anthropogenic climate change has been described as the greatest global health threat of the 21^st^ century [1] and disproportionately affects vulnerable populations [2], contributing to global injustice. It is predicted 250,000 additional deaths per year by 2030 will be attributable to climate sensitive diseases [3]. Air pollution is the biggest environmental cause of death, predicted to contribute towards seven million deaths annually [4]. Whilst planetary health impacts human health, the healthcare sector itself contributes to climate change, representing an estimated 4.4% of global greenhouse gas emissions [5]. In many nations, including the UK, health services have set targets to reach net zero carbon emissions [6, 7].

There are calls to embed climate-health and sustainability education into medical curricula, and the General Medical Council (GMC) 2018 outcomes for graduates [8] stipulate that ‘newly qualified doctors must be able to apply the principles, methods and knowledge of population health and the improvement of health and sustainable healthcare to medical practice. The Greener NHS programme identifies that staff ‘will need to be supported to learn, innovate and embed sustainable development into everyday actions’ [6]. Hence, medical students need to be prepared to address and manage the impacts of the changing environment [9] as well as to fulfil a key role in climate mitigation and health service adaptation [10]. Along with the American Medical Association which supports teaching on climate change in undergraduate curricula [11], the association for Medical Education in Europe (AMEE) recommends integration of climate-health and sustainability education and finds that graduates are not prepared for these roles [12]. In fact, globally only 15% of Medical Schools include climate change in their curricula [13]. While the Planetary Health Report Card Initiative (PHRC), an advocacy tool that allows medical schools to measure progress towards programmatic improvement, describes some progress on integration of climate change into curricula in 5 countries [14], PHRC has not yet been taken up in the rest of the world. As a result, medical students continue to call for their education to be adapted [15].

Discussions on the optimum approach to integrating climate-health and sustainability into medical curricula are ongoing [12, 16, 17] but the importance of mainstreaming education for sustainable healthcare as a transdisciplinary theme throughout undergraduate curricula has been emphasized [12, 17]. Tun et al. identified a lack of knowledgeable educators as the biggest barrier to implementation of teaching and learning on climate-health and sustainability and recommend pooling of resources (including online modules) to support delivery of teaching [16]. Walpole et al. also found lack of faculty expertise, and additionally highlighted difficulties in securing an allocation of core curriculum time and resources [17]. It has been pointed out that expert material is available [10] with organisations including the Global Consortium on Climate and Health Education (GCCHE) working to develop, pool and share resources and educational practices worldwide [18], as well as initiatives such as the PHRC providing a platform to share ideas and resources as well as evaluate and compare medical schools engagement with education of sustainable healthcare [14, 19].

Online teaching has been successfully employed in various other aspects of the medical curriculum, and meta-analyses show that students perform at least as well, or better, after online learning compared to face-to-face sessions [20, 21]. Large scale adoption of online teaching formats throughout the early stages of the COVID-19 pandemic [22] highlight the potential feasibility of these approaches. Whereas there are a number of online training platforms for climate and health [18], we do not know of any study to date that has evaluated a climate-health and sustainability module for medical students or other healthcare staff. Such a module could be a useful tool to support student learning, would not require faculty for delivery, and could be easily disseminated.

The primary aim of our study was to design and evaluate an online module aimed at 3^rd^ and 4^th^ year UK medical students entitled ‘Climate Change and Sustainability in Clinical Practice’. Secondary aims were to assess students’ attitudes towards education on themes related to climate change and sustainability in healthcare.

## Methods

### Study design

This was an uncontrolled, before and after study design to evaluate a pilot module on climate change. Students were enrolled into the E-learning module: ‘Climate Change and Sustainability in Clinical Practice’ and completed a pre-module questionnaire, post-module questionnaire, and module evaluation.

### Intervention and content

With support from experts in medical education, learning technology, and sustainable healthcare, we designed and developed an E-learning module using WordPress© software, titled: ‘Climate Change and Sustainability in Clinical Practice’ (hereafter referred to as ‘the module’). As recommended by expert groups [23], Mayer’s design principles of multimedia learning [24] were used in developing the module, as well as computer-based teaching module design principles outlined by Lau et al. [25] The module aimed to achieve learning objectives adapted from previously established priority learning outcomes [26], namely for learners to be able to describe the interactions of the environment and human health at different levels, and to develop knowledge in how to improve the environmental impact and sustainability of clinical pathways.

**Fig. 1:**
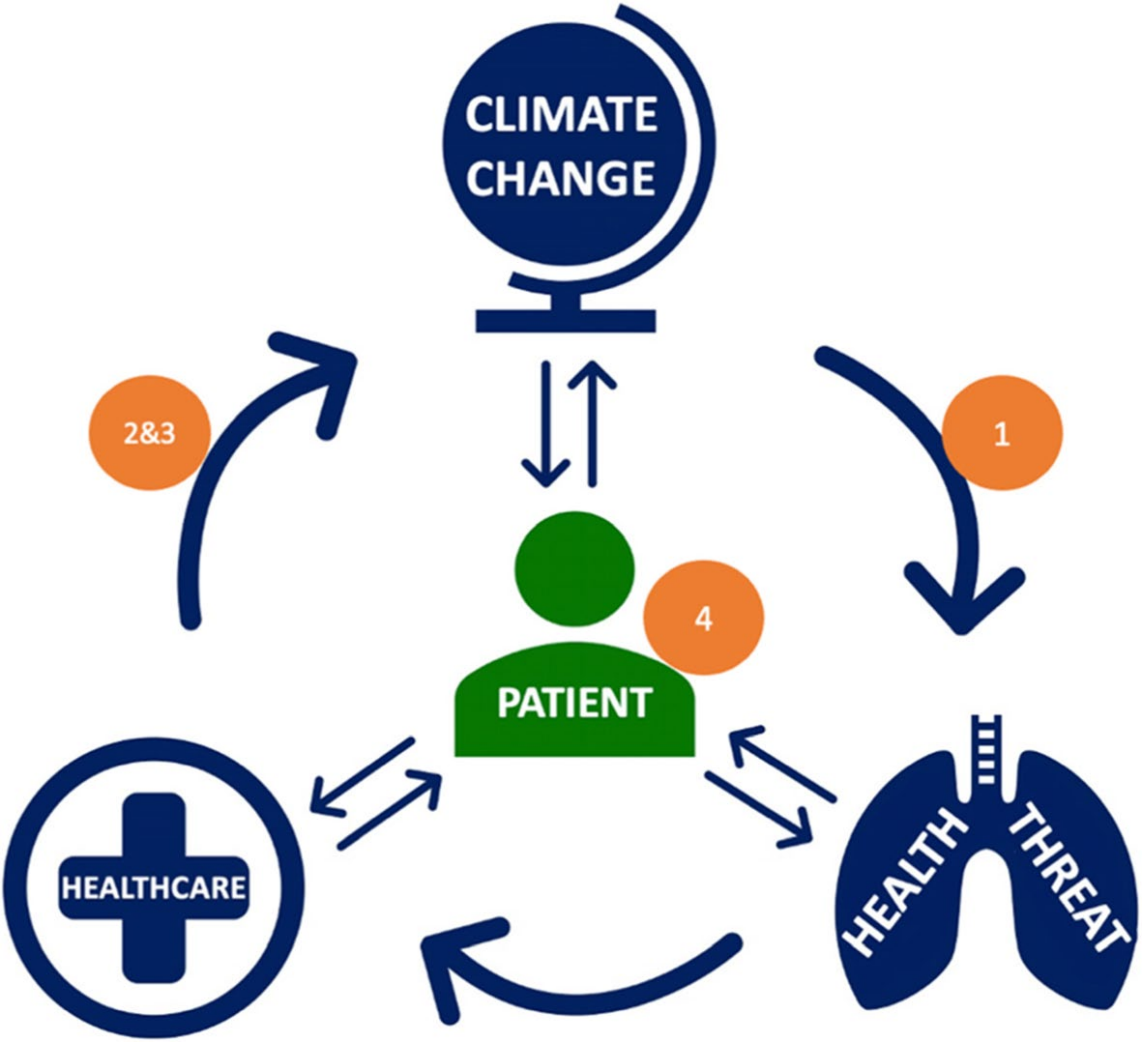
Module Infographic. Chapter 1 Climate Change: Sustainability Driver in Healthcare. Chapter 2 Environmental Impacts of Healthcare and Healthcare’s Carbon Hotspots. Chapter 3 Principles of Sustainable Clinical Practice. Chapter 4 Health Co-Benefits of Climate Mitigation

The module consists of four chapters (Fig. 1 and supplementary material 1, 2, 3, 4 and 5):

- ‘Climate Change: Sustainability Driver in Healthcare’ outlines the direct and indirect health threats of climate change, and key global and local commitments to climate mitigation.
- ‘Environmental Impacts of Healthcare and Healthcare’s Carbon Hotspots’ examines the environmental impact of health and social care in the UK, illustrated using example clinical pathways.
- ‘Principles of Sustainable Clinical Practice’ describes four previously described sustainable clinical principles [25] and invites students to consider how these can be applied in various healthcare settings.
- ‘Health Co-Benefits of Climate Mitigation’ uses case-based scenarios to examine how clinicians can advise patients to take steps that improve both their individual health and environmental impact.

Each chapter concludes with a quiz followed by detailed explanations of the answers including links to further reading. There was no grading or recording of results.

### Participants and enrolment

An email was circulated fortnightly in June and July 2020 inviting all 3^rd^ and 4^th^ year medical students (including students intercalating between their 3^rd^ and 4^th^ year) at Brighton & Sussex Medical School (BSMS) to enrol in the pilot study (*n* = 316). Students who enrolled were entered into a prize draw for two £20 vouchers, and those who completed the module were able to download a certificate.

### Survey

Online pre- and post-module surveys were developed using Qualtrics© Software with input from medical educators at University Hospitals Sussex, and BSMS. The pre- module survey used 5-point Likert scale questions to assess students’ attitudes to sustainability in healthcare, as well as subjective, self-reported understanding of four key concepts linked to the module learning objectives. These same questions were included in the post-module survey, alongside module evaluation questions to assess the ease of use and the relevance and appropriateness of modules content. Survey completion was voluntary and anonymous. (See supplementary material).

### Statistical analysis

Survey responses were converted into numerical values and tested for normality using a Shapiro–Wilk test. Differences between pre- and post-module responses regarding understanding of each of the four key concepts were assessed using Mann Whitney U tests (SPSS v26, IBM New York), with significance set at *p* < 0.05.

Thematic analysis of free text responses was performed using a general inductive approach [27]. Multiple readings were performed, and categories identified and defined by investigators. Responses within each category were coded into themes. This process was performed independently by investigator HD and TT, their analyses were compared, and final categories and themes agreed.

## Results

There were 55 responses to the pre-module questionnaire and 33 for the post module questionnaire. Of the 33 students that completed the post-module questionnaire, around two thirds of students (21/33; 63%) completed this module in one attempt, with 10 students taking two attempts, one student took three attempts, and another took four. Students were required to take the pre-module questionnaire on each attempt, therefore, 40 students enrolled onto the module (40/316 = 13% response rate). Of these, 33 completed the module and post-module surveys (33/40 = 83% completion rate).

Three of the 55 responses to the pre-module questionnaire were left blank and were excluded from analysis.

### Module performance and participant’s motivations

**Fig. 2:**
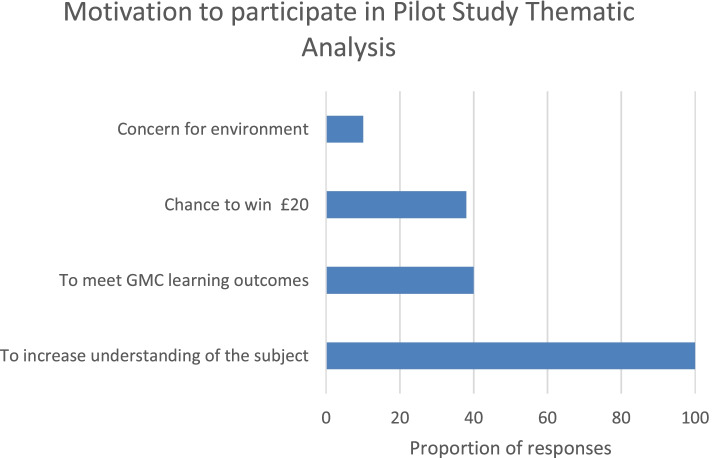
Breakdown of themes in students’ responses to the question: Why did you choose to participate in this pilot study?

The majority of students (32/33; 97%) reported completing the module within 90 min, and most frequently in 30–60 min (19/33, 58%). All students agreed that the module was: appropriate for their level of training; relevant to their training; and that ‘all students should enrol in the E-module during their training’. Almost all students (32/33; 97%) agreed that the module was easy to use. Students’ motivation to participate was assessed. Common themes in students’ responses to the question: ‘Why did you choose to participate in this pilot study?’ were identified (Fig. 2).

### Module impact on student reported understanding of key themes

The post-module evaluation demonstrated a significant increase in understanding of each of the four key concepts related to climate change, health, and sustainability in healthcare (*p* < 0.001) Table 1. Pre-module understanding of each theme was rated as good 1/52 (2%) or excellent by 11/52 (21%) students, this improved to between 30 – 32/52 (91 – 97%) students after completion of the module. Prior to undertaking the module ‘the environmental impact of clinical pathways’ was the least well understood theme with most students (41/52; 79%) reporting terrible or poor understanding. After the module no students reported terrible or poor understanding and 31/33 (94%) reported good or excellent understanding.

**Table 1:** Pre- and Post-Module student reported understanding of key themes

Please rate your understanding of:	Survey*	Terrible n (%)	Poor n (%)	Average n (%)	Good n (%)	Excellent n (%)
The environmental impact of clinical pathways	Pre	11 (21)	30 (58)	10 (19)	1 (2)	0 (0)
	Post	0 (0)	0 (0)	2 (6)	26 (79)	5 (15)
The influence of climate change on health	Pre	3 (6)	19 (37)	19 (37)	10 (19)	1 (2)
	Post	0 (0)	0 (0)	1 (3)	20 (60)	12 (36)
The principles of sustainable clinical practice	Pre	10 (19)	25 (48)	16 (30)	1 (2)	0 (0)
	Post	0 (0)	0 (0)	3 (9)	22 (67)	8 (24)
The health co-benefits of climate action	Pre	6 (12)	20 (38)	15 (29)	10 (19)	1 (2)
	Post	0 (0)	0 (0)	1 (3)	19 (58)	13 (39)

^* ^The pre-module survey number (n) is out of 52, the post module survey is out of 33

### Module impact on attitudes towards sustainability and healthcare

Prior to completing the module, the majority of the students felt that they had received ‘far too little’ (28/52; 54%), or ‘too little’ (22/52; 42%) teaching on climate change and health. On completion of the module all students felt that both ‘understanding the implication of climate change on health and healthcare’, and ‘improving the sustainability of clinical practice’ were very important. Almost all students (32/33; 97%) agreed with the statement ‘healthcare workers have a responsibility to reduce the environmental impact of the NHS’.

### Module evaluation white space answers

**Fig. 3:**
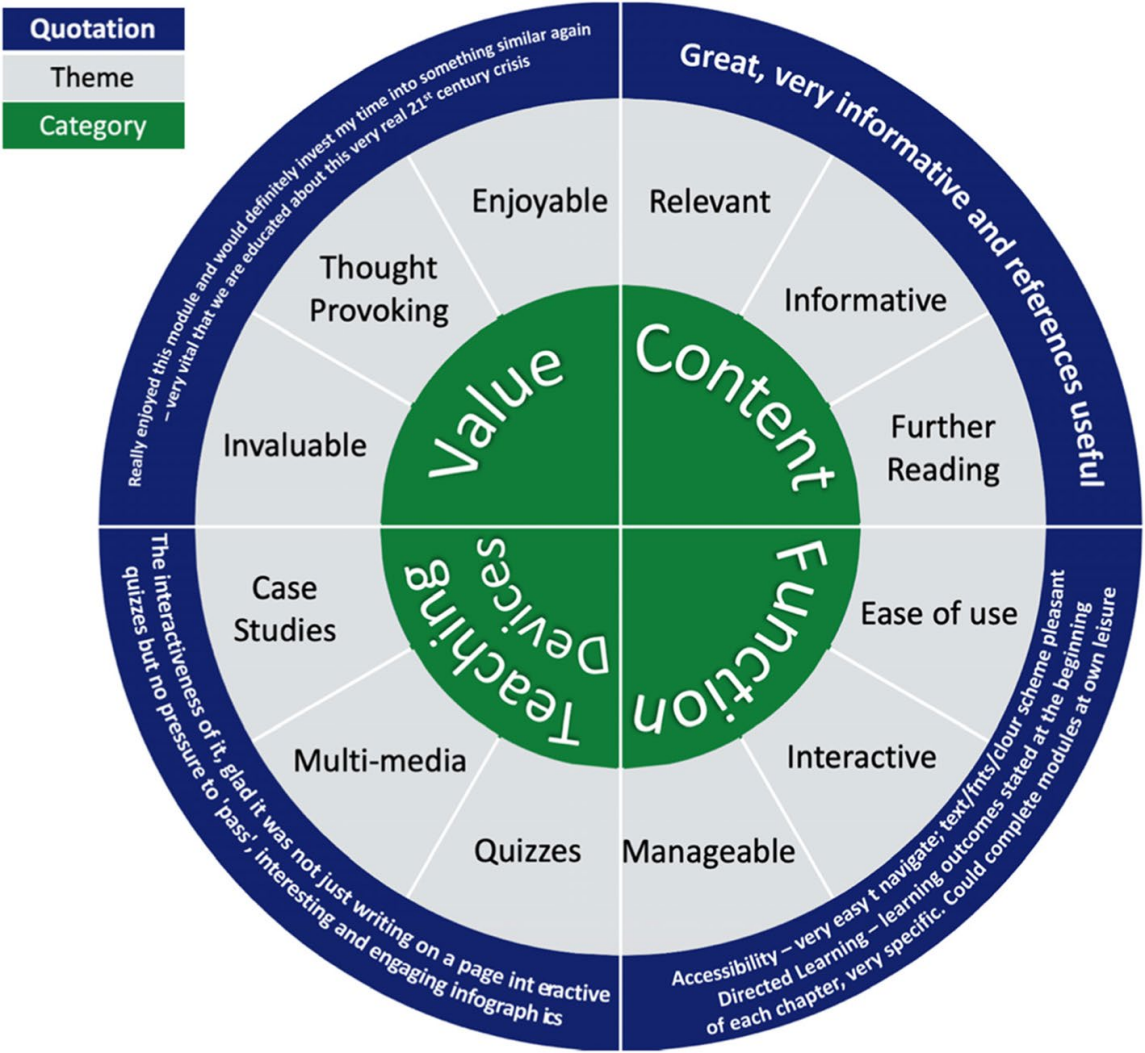
Module evaluation Thematic analysis: What did you like the most about this module?’ & ‘Any other comments’

Free text responses to white space questions were almost entirely positive. Responses to the questions ‘What did you like the most about this module?’ and ‘Any other comments’ were classified into four categories: function, value, content, and teaching devices (Fig. 3). Common themes included positive comments surrounding the module interactivity and use of integrated videos, as well as students highlighting the importance of the topics covered and perceived relevance to their training.

**Fig. 4:**
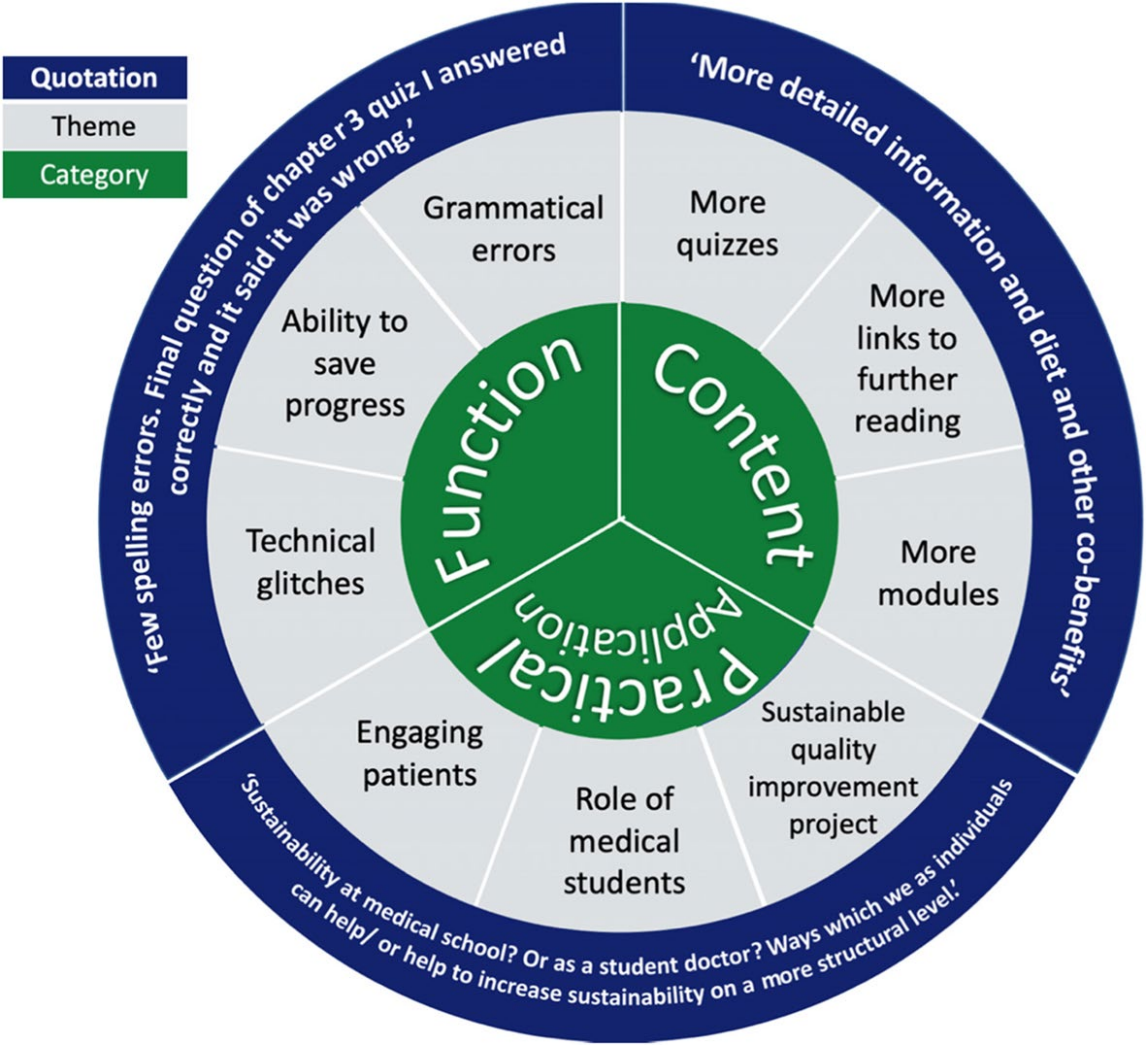
Module evaluation Thematic analysis: ‘What things could be improved?’ and ‘What other teaching of sustainable healthcare would you value?. Legend: wheel infographics that present categories and themes identified and agreed by investigators in the thematic analysis of student’s feedback. Categories, into which responses were coded are represented in the centre circle of the wheel (green) The three most significant themes within each category are displayed in the second circle (grey). A direct quotation from student’s responses coded into relevant categories is displayed in the outer circle (blue)

Responses to the questions ‘What things could be improved?’ and ‘What other teaching of sustainable healthcare would you value?’ were classified into three categories: function, content, and practical application (Fig. 4). Here we found students commonly reported a desire for more resources related to the topics covered, as well as further guidance on how to make practical steps towards achieving a net-zero health service. Students indicated that their user experience would have been improved through inclusion of a ‘go back’ button within quizzes, and we have been able to implement this.

## Discussion

Here we describe development of an online self-directed learning resource which sought to introduce medical students to important concepts in climate health and sustainability, aligned with identified priority learning outcomes [26, 28]. To our knowledge, this is the first study to evaluate learner outcomes of an online module in the field of sustainable health and healthcare. Student feedback indicated good module usability and applicability, and our results suggest that completion of the module was associated with significant improvement in self-assessed knowledge of key concepts in climate health and sustainability.

Our findings also support the notion that medical students recognise the importance of climate-health and sustainability but feel that their current curriculum does not include sufficient teaching on the topic [29]. Our module sought to address this, takes around 1.5 h to complete, and because it is delivered online and does not require physical resources or expert faculty for its delivery, can easily be integrated into medical curricula.

Several students commented that they would value teaching on practical applications of the modules content, including how individuals can make a difference to sustainability of healthcare, and this would add value to future iterations of this module. As understanding of key concepts in health and climate change increases amongst healthcare professionals, this should be aligned with teaching and learning in sustainable quality improvement to support real-world action [26, 30].

The module has been further developed based on student feedback provided from this study and is being integrated into the undergraduate curriculum at BSMS medical school. The online module will be available to students to supplement their learning alongside the sustainability in healthcare modules which are incorporated throughout the curriculum [31]. We intend to expand access to the module to support learning on climate change and sustainable clinical practice amongst healthcare professionals at our local hospitals. As knowledge in this field is relatively independent of clinical seniority, this module may also prove be helpful at a postgraduate level. This aligns with plans by the team at the Greener NHS [6] for an online module to build sustainable development knowledge and capability in all healthcare staff.

There are some limitations to this study. We did not perform any formative assessment to assess understanding of key themes, and instead have interpreted students perceived improvement of understanding. We did not receive the same number of pre- and post- module responses, which limited some analyses. Despite this, differences between pre- and post-module scores were significant at *p* < 0.001. The questionnaire method was limited by the fact that students who took more than one attempt to complete the module had to complete the pre-module questionnaire on each attempt. The low response rate indicates probable selection bias, and it is likely that students who are interested in the topic were more likely to participate. However, almost four fifths of participants rated their understanding of each of four key concepts related to climate change and sustainable clinical practice as worse than average. This reported low knowledge even amongst those predisposed to such learning further supports the need to develop more teaching and learning on this topic.

We hope that our approach is emulated elsewhere, as this will help to support making delivery of health and healthcare more environmentally sustainable.

## Supplementary Information

**Additional file 1.**

**Additional file 2.**

## Data Availability

The datasets used and/or analysed during the current study are available included in this published article and its supplementary information files.

## Abbreviations

NHS: National Health Service
GMC: General Medical Council
BSMS: Brighton and Sussex Medical School
AMEE: Association for Medical Education in Europe
GCCHE: Global Consortium on Climate and Health Education
PHRC: Planetary Health Report Card
