# Supplementary material for: Effectiveness of an online module: climate-change and sustainability in clinical practice
Source: BMC Med Educ. 2022 Sep 17;22:682. doi: 10.1186/s12909-022-03734-8 (PMC9482263; doi:10.1186/s12909-022-03734-8)
Supplement: Supplementary file 1 — Additional file 1. [file 12909_2022_3734_MOESM1_ESM.docx]

Supplementary material

1. Home page:


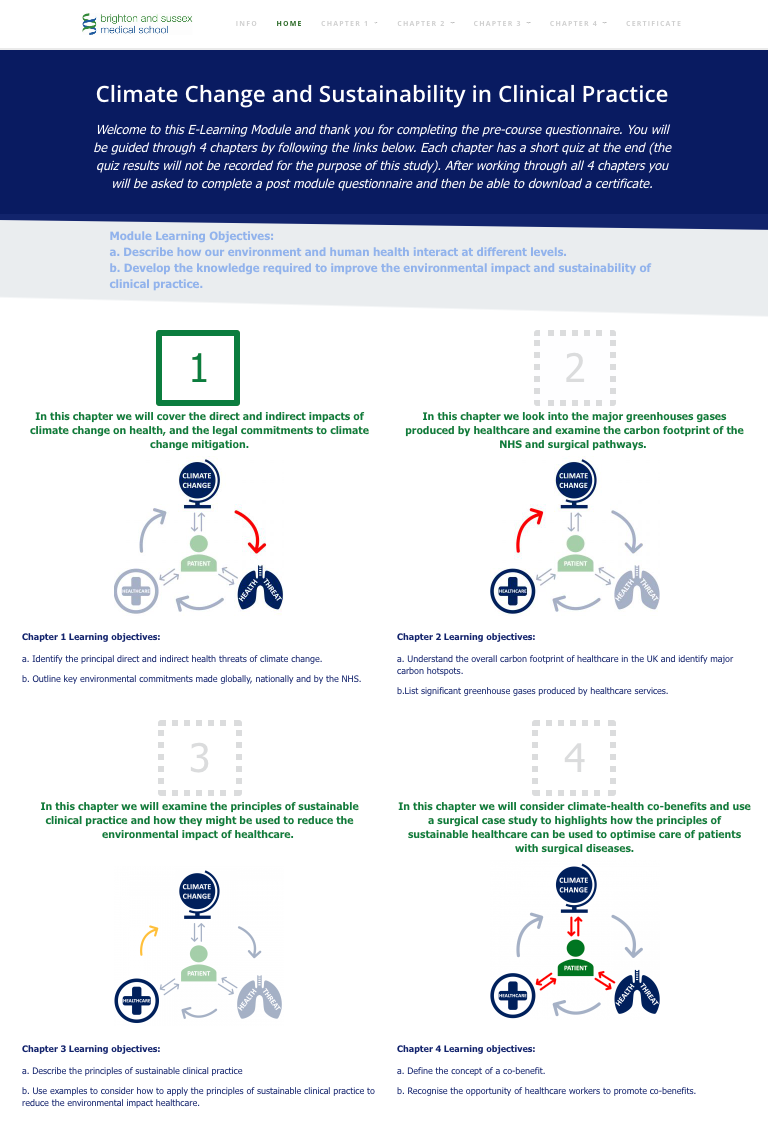


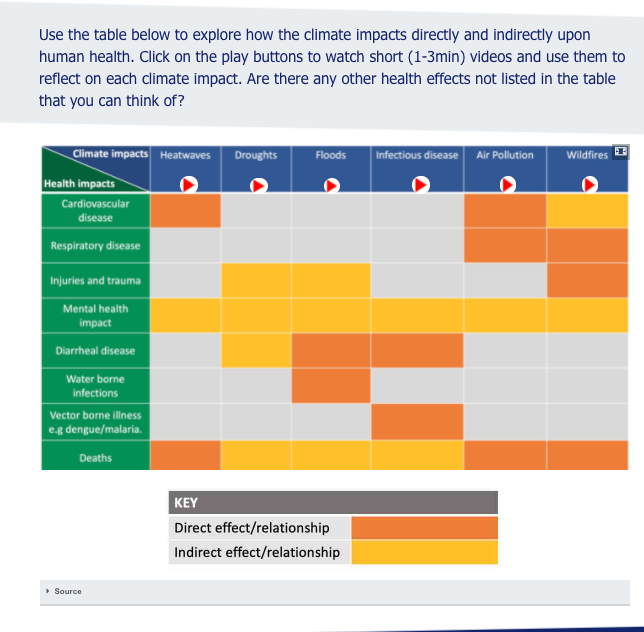

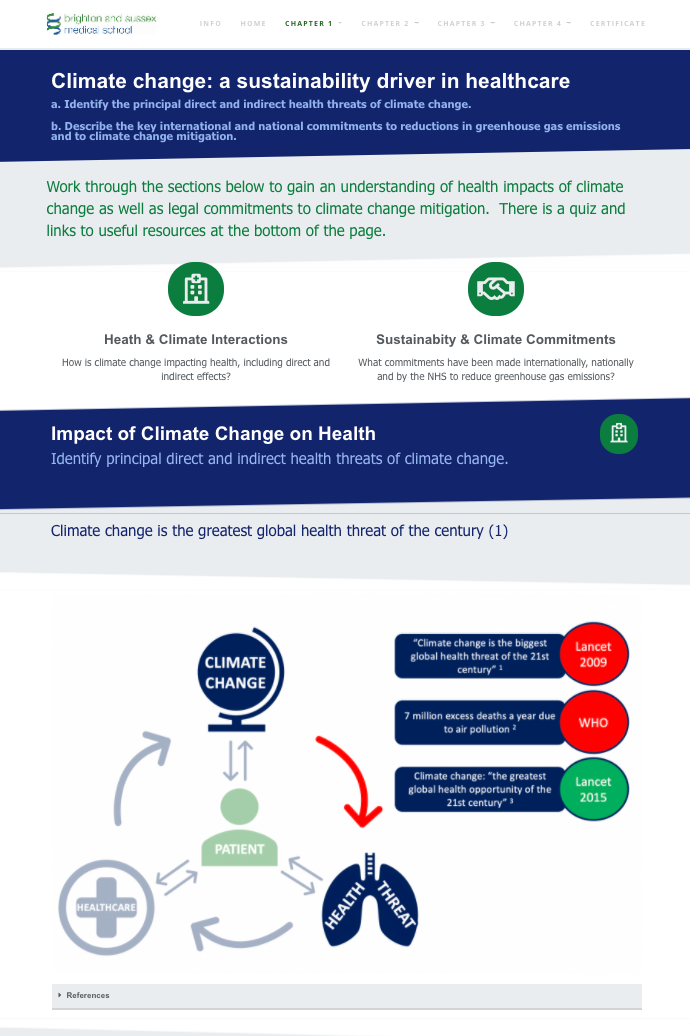
2. Chapter 1


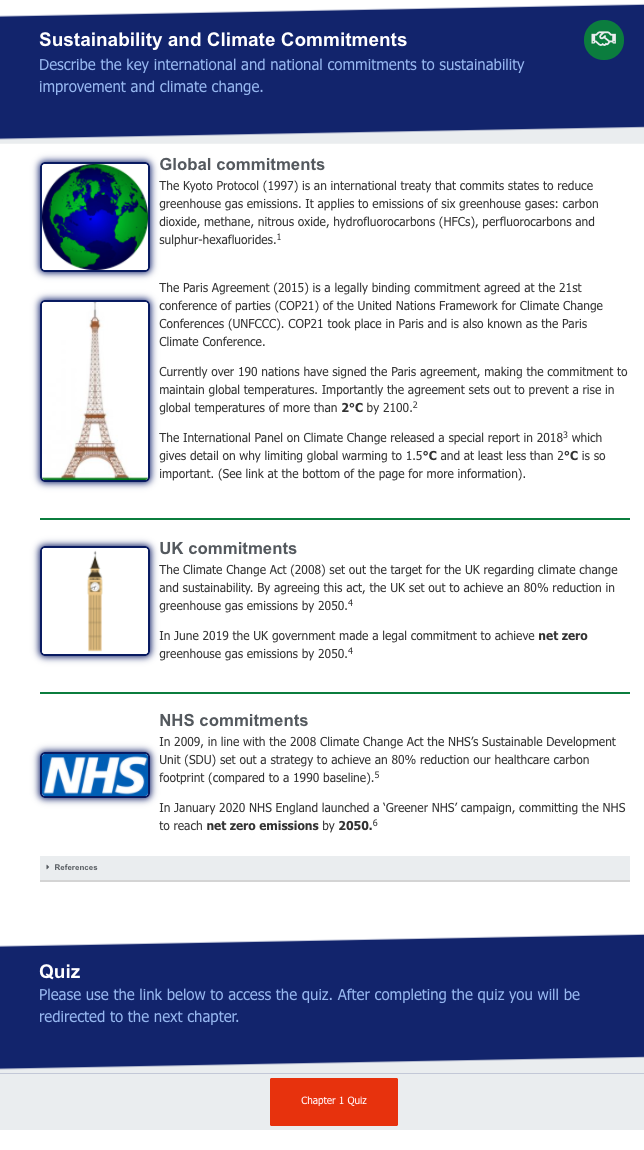


3. Chapter 2:


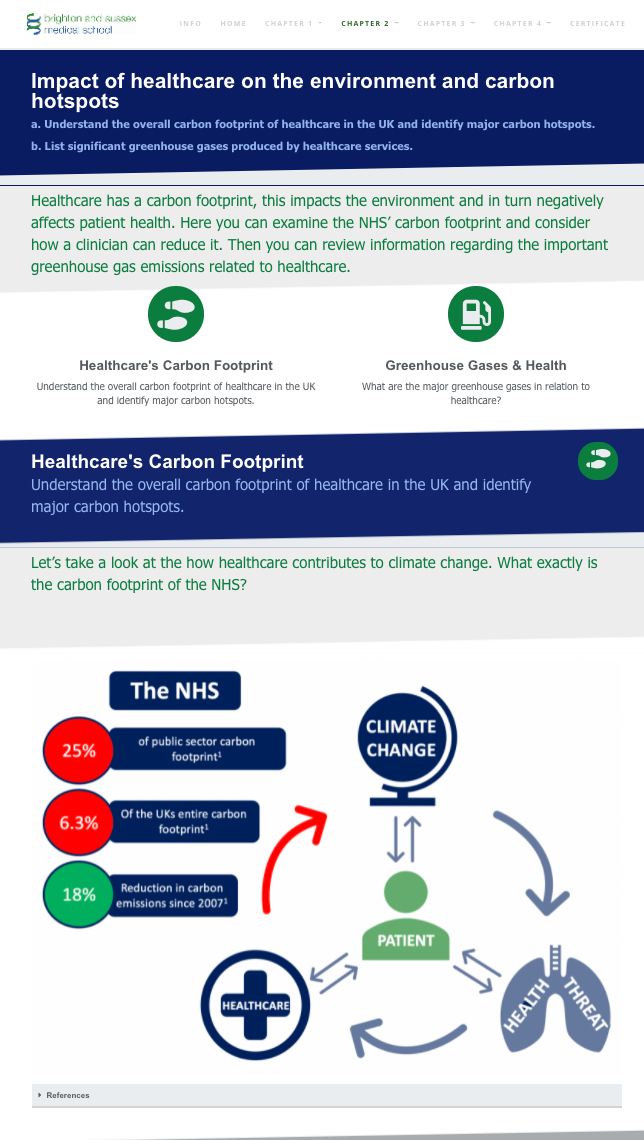


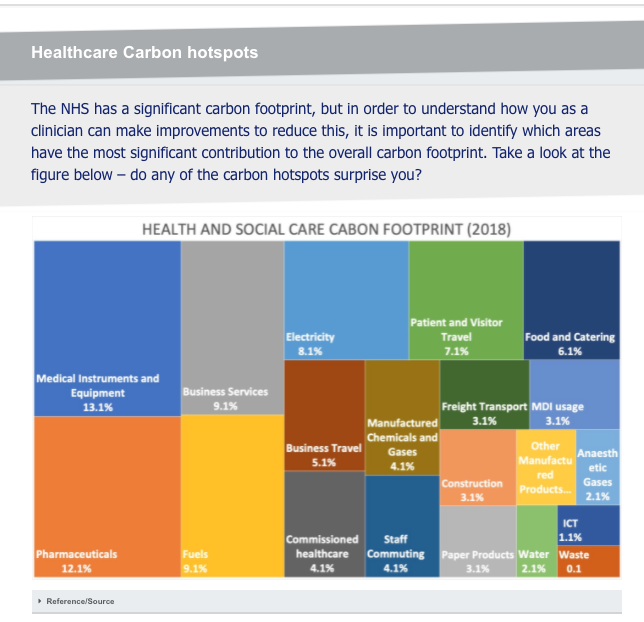


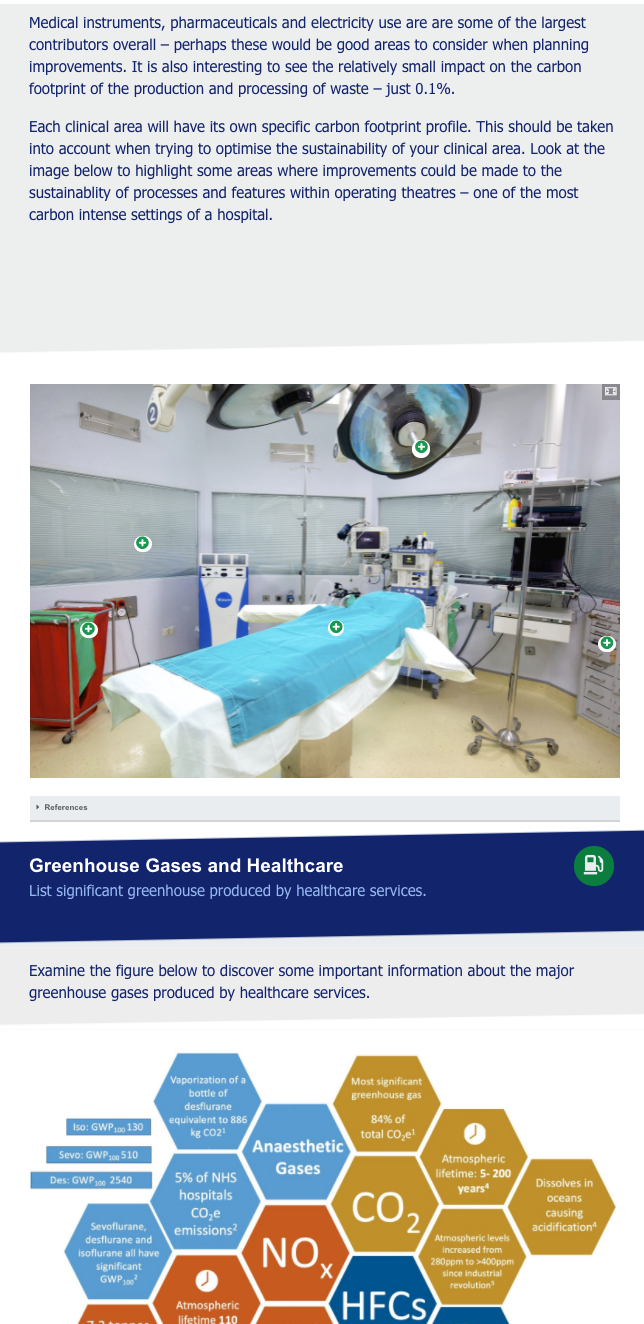


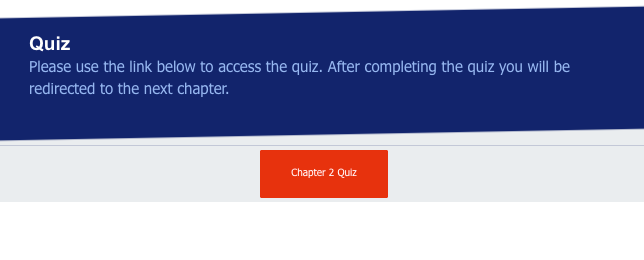


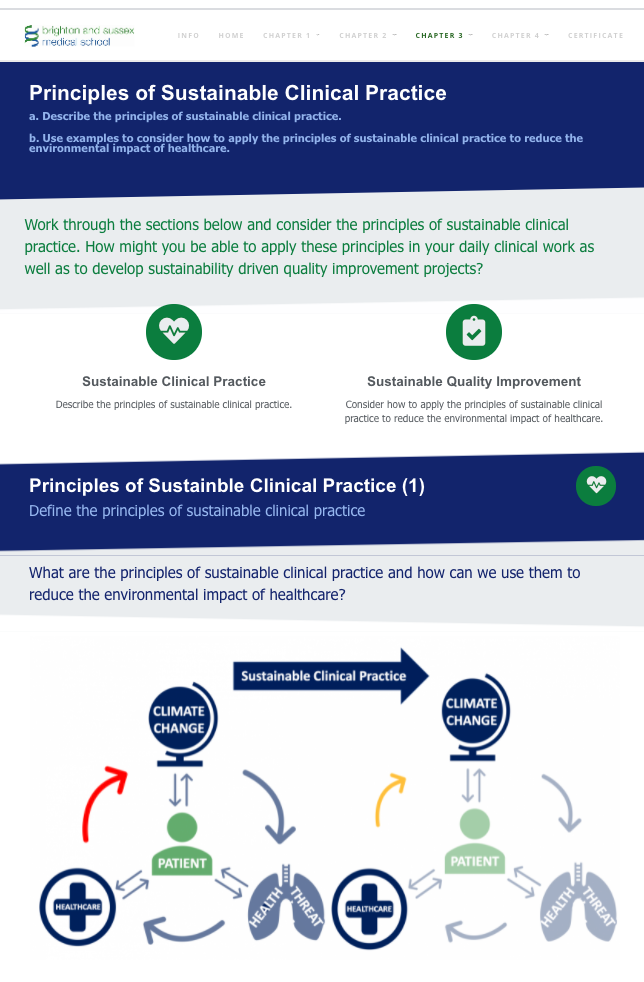
4. Chapter 3.


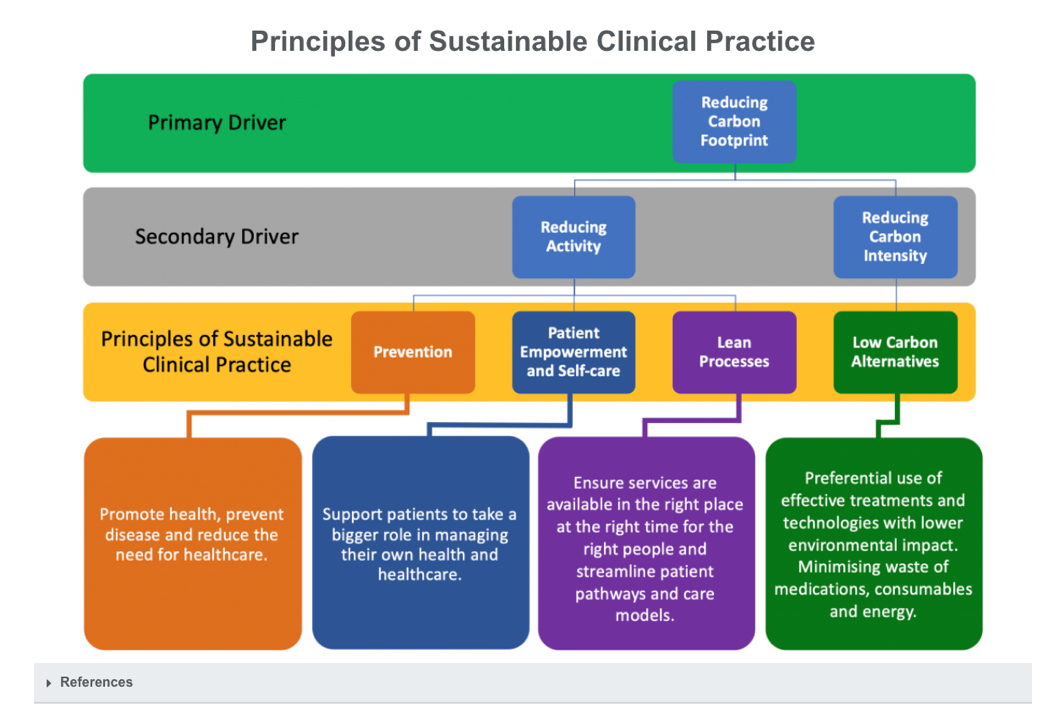


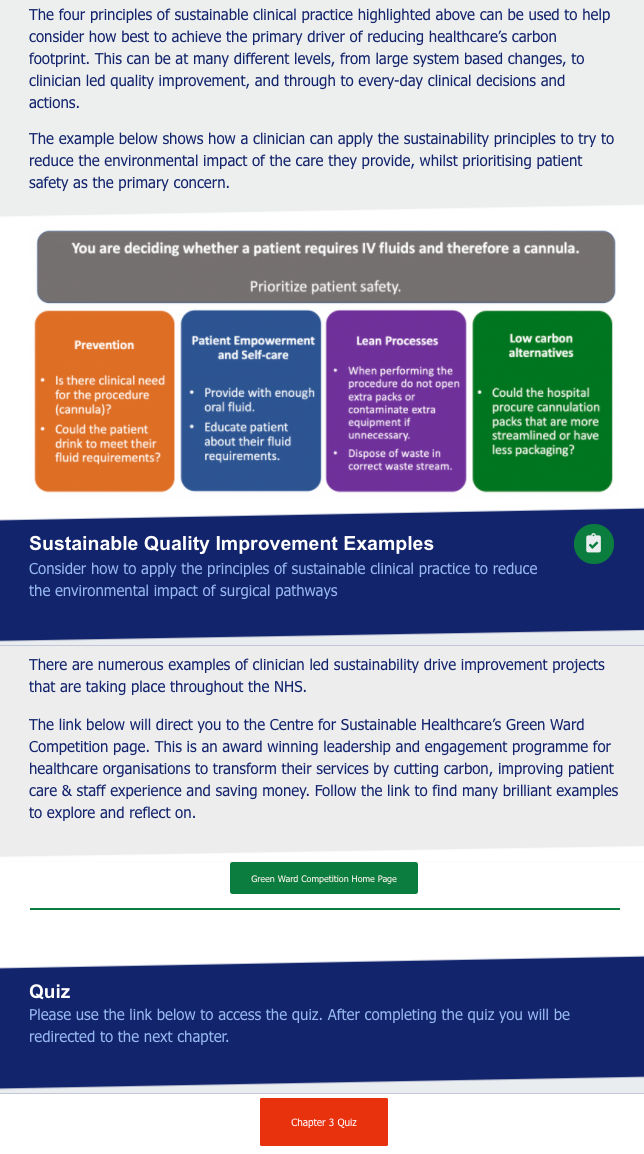


5. Chapter 4.


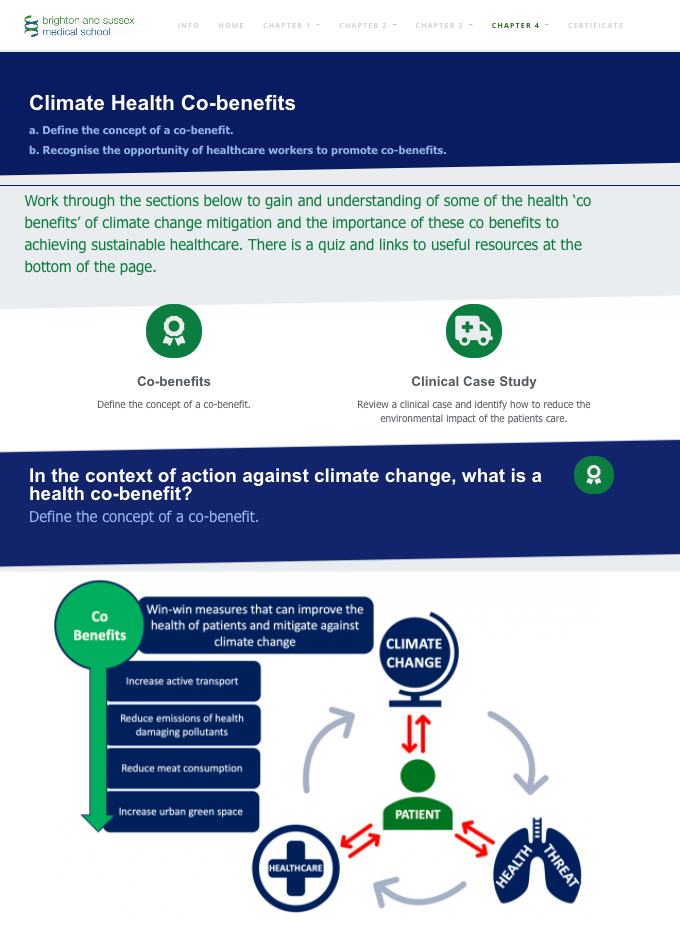


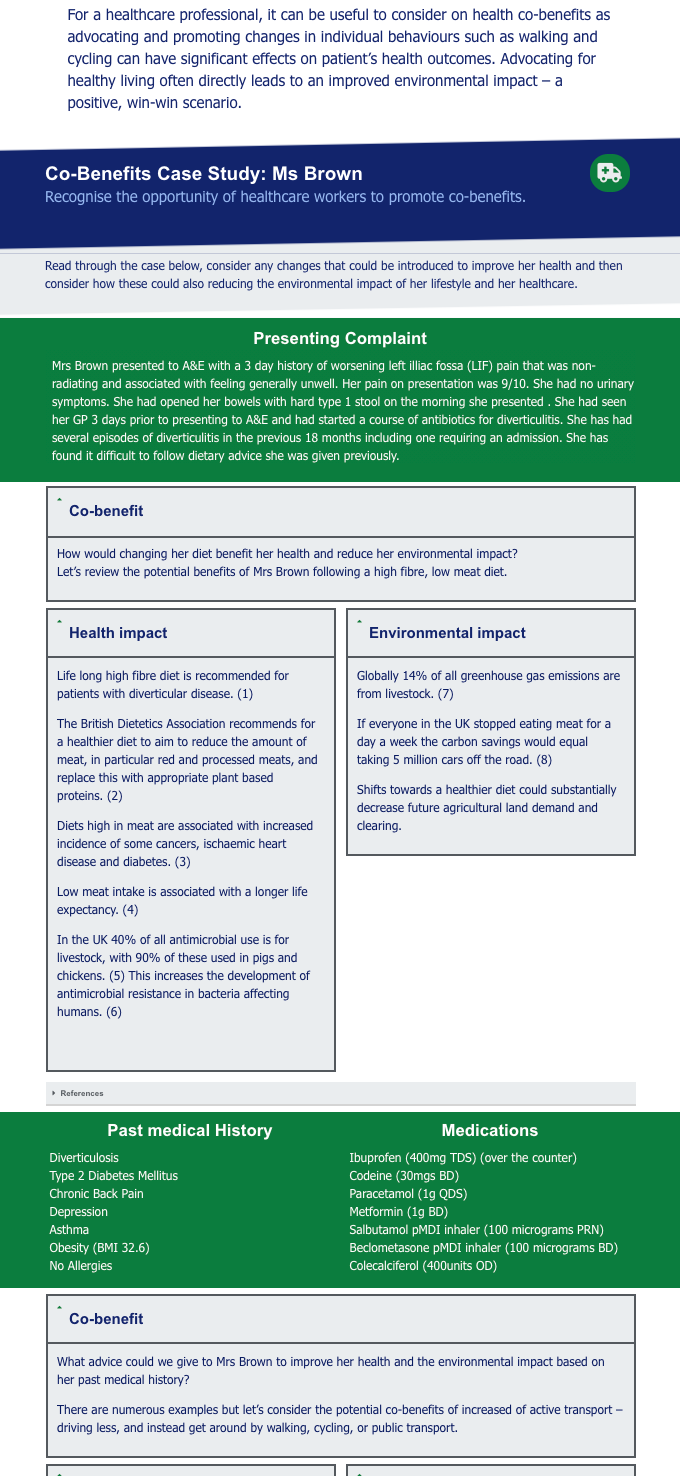


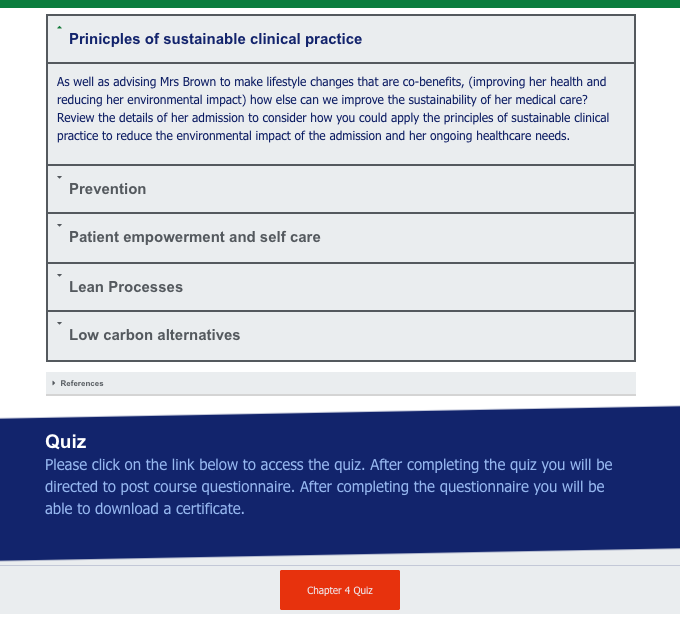


**5. – Questionnaires:**

Climate change and Sustainability in Clinical Practice: Pilot Study Questionnaire

Anonymised questionnaire using Qualtrics online survey tool to include free text and scaling questions.

Parts 1 and 2 of the questionnaires will form the pre-module and the post-module questionnaire.
Part 3 of this questionnaire will form the module evaluation and will be included in only the post-module questionnaire.

Participants will be unable to progress to the questionnaire unless they have completed the consent form.

**Part 1: Participant Demographics + Motivation**Q1: What year of medical school are you currently in? 3^rd^ year/4^th^ year/other.
Q2: Gender? F/M/Prefer not to say.
Q3: Currently what is your medical career preference? General Practice/Medical Specialty/Paediatrics/Psychiatry/Surgical Specialty/I do not yet know/Other]
Q4: Why have you chosen to participate in this study? [free text]

**Part 2: Pre- and Post-module questionnaire**

Q5: How much teaching on climate change and health have you received so far during medical school?
(Far too much/Too much/ Neither too much nor too little/Too little/Far too little)

Q6:Please rate the importance of the following statements:
Understanding the implications of climate change on health and healthcare is:
Improving the sustainability of clinical practice is:
(Extremely important/Very important/Moderately important/Slightly important/Not at all important.)

Q7 Healthcare workers have a responsibility to reduce the environmental impact of the NHS. To what extend do you agree?
(Strongly agree/Somewhat agree/Neither agree nor disagree/Somewhat disagree/Strongly disagree.)

Q8: Please rate your understanding of the following concepts:
The environmental impact of clinical pathways
The influence climate change has on health
The principles of sustainable clinical practice
The health co-benefits of climate action.
(Excellent/Good/Average/Poor/Terrible)

Q9: In the context of health care, what does the term sustainability mean to you? [Free text]

**Part 3 Module evaluation**Q10: How long did this module take you to complete?

Q11: In how many sittings did you complete the module?

Q12: To what extend do you agree with the following statements?
The content of the E-module was at an appropriate level for my training.
The content of the E-module was relevant to your training.
The online module was easy to use.
All medical students should undertake this module as part of their training.
(Strongly agree/Somewhat agree/Neither agree nor disagree/Somewhat disagree/Strongly disagree)

Q13: What did you like most about the E-module? [Free text]

Q14: What were the things that could be improved [Free text]

Q15: What other learning on sustainable healthcare would you value? [Free text]

Q16: Any other comments [Free text]
